# Draft genome sequence including the capsule operon of a *Bacillus cereus* strain isolated from a patient with bacteremia in Japan

**DOI:** 10.1128/MRA.00766-23

**Published:** 2024-01-05

**Authors:** Akiko Okutani, Shu Okugawa, Fumie Fujimoto, Mahoko Ikeda, Takeya Tsutsumi, Kyoji Moriya, Ken Maeda

**Affiliations:** 1 Department of Veterinary Science, National Institute of Infectious Diseases, Tokyo, Japan; 2 Department of Infectious Diseases, The University of Tokyo Hospital, Tokyo, Japan; 3 Department of Infection Control and Prevention, The University of Tokyo Hospital, Tokyo, Japan; Department of Biological Sciences, Wellesley College, Wellesley, Massachusetts, USA

**Keywords:** *Bacillus cereus*, capsule, bacteremia

## Abstract

*Bacillus cereus*, which causes opportunistic infections in hospitals as well as food poisoning, is genetically similar to *Bacillus anthracis*. We herein report the draft genome including the capsule operon of *B. cereus* BCER1 isolated from the blood of a hospital patient in Japan.

## ANNOUNCEMENT

**Fig 1:**
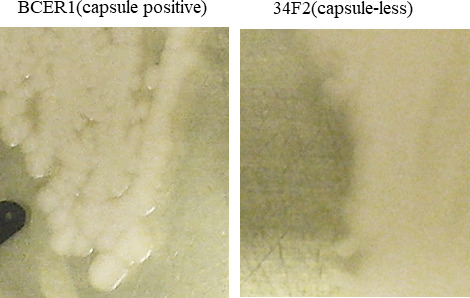
Shiny, blight, and moist surface of BCER1 colonies expressing capsule and dry surface of uncapsulated 34F2 colonies on bicarbonate agar are shown.

*Bacillus cereus* is an opportunistic pathogen that causes various infections, including local infections of wounds (1), bacteremia and septicemia (2), and central nervous system infections (3). We previously reported the assembly of the complete genomes of three *B. cereus* isolates (GTC2903, GTC2926, and ach14) derived from hospital patients in Japan; of these isolates, GTC2926 lacks the complete capsule operon (4, 5). To clarify the genetic background and virulence gene profiles of *B. cereus* clinical isolates in Japan, we screened 28 other *B. cereus* isolates from patients aged 10–89 years with bacteremia caused by *B. cereus* in the University of Tokyo Hospital. Blood was added to BACTEC standard culture bottles, which were then incubated in a BACTEC 9000 system (Becton, Dickinson and Company, Franklin Lakes, NJ, USA) at 37°C for 24 h. The isolates were stored as frozen stock. All isolates were identified as *B. cereus* using the VITEK2 system with the BCL identification card (SYSMEX bioMerieux, Tokyo, Japan) (6) and the matrix-assisted laser desorption/ionization time-of-flight mass spectrometry system generating a mass spectrum pattern of *B. cereus* with MALDI Biotyper (version 2.0) (Bruker Daltonics, Bremen, Germany) according to the manufacturer’s instruction. One strain, BCER1, which was isolated in 2005 from a patient with bacteremia, produced a capsule on bicarbonate agar medium; its colony surface was moist, shiny, and bright, which was in contrast to the dry colony surface of the capsule-less strain 34F2 (Fig. 1). We amplified the complete *capB* gene in the capsule operon of the BCER1 genome by PCR according to the World Health Organization manual (7). Additionally, long-read genome sequencing was performed to determine whether the BCER1 genome contains the complete capsule operon and where in the genome this operon was inserted.

Each culture from the frozen stock was incubated in Luria–Bertani broth at 37°C for 24 h. Genomic DNA was extracted from the broth culture using the QIAamp DNA Mini Kit (Qiagen, Hilden, Germany). A 15–18 kb library was prepared using the Single-Molecule Real-Time (SMRT) Cell Template Prep kit (PacBio, California, USA) and then sequenced using the PacBio Sequel II system (PacBio) (8). After filtering, 5,572,927 bp was assembled with an *N*_50_ value of 5,507,456 bp, mean length of 1,114,585 bp, and G+C content of 35.3% using the default parameters of the microbial genome assembly application in SMRT Analysis (version 12.0) (http://www.pacb.com/products-and-services/pacbio-systems/sequel). Mapping reads against the assembled contigs and polishing using Racon (original version) (9) resulted in five contigs. The sequences were annotated using the rapid prokaryotic genome annotation tool DFAST (version 1.2.0) (10). Contig details are provided in Table 1.

**TABLE 1:** Genome assembly metrics and genetic features of BCER1 contigs

Contig name	Total size (bp)	G + C content (%)	putative coding sequences (CDS)	tRNA genes	rRNA genes
Contig 1	5,507,456	35.3	5515	107	39
Contig 2	40,391	35	35	-^*a*^	-
Contig 3	12,829	36.2	13	-	-
Contig 4	9,160	31.4	11	-	-
Contig 5	3,091	34.9	2	-	-

^*a*^ -, not detected.

## Data Availability

The raw reads of *Bacillus cereus* strain BCER1 have been deposited in DDBJ/GenBank under the accession number DRA017100 and the genome sequence under the accession numbers BSWH01000001, BSWH01000002, BSWH01000003, BSWH01000004, and BSWH01000005.

## References

[1] Acosta Pedemonte NB, Rocchetti NS, Villalba J, Lerman Tenenbaum D, Settecase CJ, Bagilet DH, Colombo LG, Gregorini ER. 2020. Bacillus cereus bacteremia in a patient with an abdominal stab wound. Rev Argent Microbiol 52:115–117. doi:10.1016/j.ram.2019.07.003 31791818

[2] Uchino Y, Iriyama N, Matsumoto K, Hirabayashi Y, Miura K, Kurita D, Kobayashi Y, Yagi M, Kodaira H, Hojo A, Kobayashi S, Hatta Y, Takeuchi J. 2012. A case series of Bacillus cereus septicemia in patients with hematological disease. Intern Med 51:2733–2738. doi:10.2169/internalmedicine.51.7258 23037464

[3] Brouland JP, Sala N, Tusgul S, Rebecchini C, Kovari E. 2018. Bacillus cereus bacteremia with central nervous system involvement: a neuropathological study. Clin Neuropathol 37:22–27. doi:10.5414/NP301041 29035192

[4] Okutani A, Inoue S, Morikawa S. 2019. Draft genome sequences of three clinical strains of Bacillus cereus isolated from human patients in Japan. Microbiol Resour Announc 8:e00415-19. doi:10.1128/MRA.00415-19 31072885 PMC6509534

[5] Okutani A, Inoue S, Noguchi A, Kaku Y, Morikawa S. 2019. Whole-genome sequence-based comparison and profiling of virulence-associated genes of Bacillus cereus group isolates from diverse sources in Japan. BMC Microbiol 19:296. doi:10.1186/s12866-019-1678-1 31842760 PMC6915864

[6] Ikeda M, Yagihara Y, Tatsuno K, Okazaki M, Okugawa S, Moriya K. 2015. Clinical characteristics and antimicrobial susceptibility of Bacillus cereus blood stream infections. Ann Clin Microbiol Antimicrob 14:43. doi:10.1186/s12941-015-0104-2 26370137 PMC4570458

[7] World Health Organization. 1998. Guidelines for the surveillance and control of anthrax in humans and animals. principal author, P. C. B. Turnbul. https://apps.who.int/iris/handle/10665/59516.

[8] Chin C-S, Alexander DH, Marks P, Klammer AA, Drake J, Heiner C, Clum A, Copeland A, Huddleston J, Eichler EE, Turner SW, Korlach J. 2013. Nonhybrid, finished microbial genome assemblies from long-read SMRT sequencing data. Nat Methods 10:563–569. doi:10.1038/nmeth.2474 23644548

[9] Vaser R, Sović I, Nagarajan N, Šikić M. 2017. Fast and accurate de novo genome assembly from long uncorrected reads. Genome Res 27:737–746. doi:10.1101/gr.214270.116 28100585 PMC5411768

[10] Tanizawa Y, Fujisawa T, Nakamura Y. 2018. DFAST: a flexible prokaryotic genome annotation pipeline for faster genome publication. Bioinformatics 34:1037–1039. doi:10.1093/bioinformatics/btx713 29106469 PMC5860143

